# Outcomes and complications of hemodialysis in patients with renal cancer following bilateral nephrectomy

**DOI:** 10.1515/med-2024-1009

**Published:** 2024-08-23

**Authors:** Bing Shen, Feng Luo, Nan Yuan, Jiaming Yin, Yalin Chai, Lijie Sun, Lin Zhang, Congjuan Luo

**Affiliations:** Qingdao University, Qingdao 266071, P.R. China; The Affiliated Hospital of Qingdao University, Qingdao 266000, Shandong, P.R. China; The Affiliated Hospital of Qingdao University, No. 16, Jiangsu Road, Shinan District, Qingdao 266000, Shandong, P.R. China

**Keywords:** bilateral nephrectomy, hemodialysis, hypotension, anemia, tumor recurrence, erythropoietin-stimulating agent

## Abstract

**Objectives:**

The management of patients undergoing bilateral nephrectomy for renal cancer presents significant challenges, particularly in addressing hypotension, anemia, and tumor recurrence during hemodialysis.

**Case presentation:**

A patient diagnosed with renal clear cell carcinoma in 2009 was followed until his demise in June 2022, with detailed documentation of symptoms, signs, laboratory results, diagnosis, and treatment. In the presented case, post-nephrectomy, the patient experienced frequent hypotension and anemia during dialysis, improving with erythropoietin-stimulating agents and subsequently with rosuvastatin. Later, multiple metastases were detected, correlating with normalized blood pressure and hemoglobin.

**Literature review:**

A literature search up to September 2023 was also conducted, gathering data on hypotension, anemia, and tumor recurrence post-nephrectomy. Literature analysis of six cases revealed a 100% tumor recurrence rate in elderly patients (>50 years).

**Conclusion:**

Treatment of anemia in bilateral nephrectomy patients warrants consideration of medication-induced tumor recurrence, highlighting early kidney transplantation to avoid adverse reactions like hypotension.

## 1 Introduction

Renal carcinoma represents a critical concern within global health domains, with its incidence and mortality rates demonstrating a rising trajectory, as substantiated by contemporary research findings [1–3]. Bilateral nephrectomy stands out as a pivotal treatment for cases presenting with locally advanced or bilateral manifestations of the disease, aiming primarily at remission achievement. Notably, this surgical approach is instrumental in the obliteration of the tumor mass, significantly enhancing patient survival prospects and quality of life [4–7]. Nonetheless, the consequent renal function forfeiture necessitates reliance on dialysis for life sustenance post-surgery [8].

Patients subjected to bilateral nephrectomy frequently face a spectrum of postoperative challenges, among which hypotension and anemia are prevalently reported [9,10]. These issues are particularly magnified during dialysis, which is essential for maintaining life postoperatively [11–13]. Hypotension exacerbates cardiovascular strain, potentially catalyzing severe outcomes such as arrhythmias and heart failure [14–16], whereas anemia significantly detracts from quality of life through sustained fatigue and weakness [17–19]. Moreover, dialysis carries inherent risks, including heightened infection and pneumonia rates, complicating postoperative management [20,21].

In the management of post-nephrectomy anemia, clinicians often turn to erythropoiesis-stimulating agents (ESAs) and darbepoetin, which are crucial in mitigating symptoms by promoting red blood cell genesis, thus restoring patient normalcy [22,23]. While these agents effectively reduce anemia, their use is not without potential side effects and implications for long-term health [24,25]. Recent studies have begun to uncover a potential association between erythropoietin and other related drug treatments with tumor recurrence and progression. This relationship exhibits a double-edged sword effect, wherein these drugs are effective in controlling symptoms of anemia but may simultaneously increase the risk of tumor recurrence [26,27]. Due to the lack of comprehensive clinical data and the limited number of case samples, the relationship between erythropoietin and tumor recurrence risk remains ambiguous. This situation underscores the need for more careful consideration and balance when selecting treatment plans. Consequently, there is an urgent need for further research to explore the impacts and mechanisms of these drugs in greater depth. This will help identify safer and more effective treatment strategies that ensure patients receive necessary care without increasing other health risks.

This investigation seeks to elucidate the complex interplay between the management of complications following bilateral nephrectomy and the resurgence of tumor risk. Through meticulous patient case examinations and a comprehensive literature review, it endeavors to enrich our comprehension and refine therapeutic methodologies.

## 2 Materials and methods

### 2.1 Clinical patient information collection

In 2009, a male patient aged 57 was diagnosed at our institution with left renal clear cell carcinoma, having presented with bilateral lumbar pain for 3 months. Based on this diagnosis, a series of monitoring procedures were initiated to assess the patient’s health status and treatment responses over time. A comprehensive set of medical records was systematically compiled. This dataset included, but was not limited to, outcomes of clinical examinations related to renal function and cancer diagnosis, surgical interventions, pathological findings, detailed records of pharmacological treatments, including drug dosages and patient responses, as well as ongoing assessments of blood pressure and hemoglobin levels. The collection process was governed by stringent quality control measures to ensure the integrity and completeness of the gathered information.

### 2.2 Clinical data processing and analysis

The collected data were meticulously organized and categorized to facilitate detailed analysis. This process focused on various critical junctures in the patient’s treatment timeline, such as the post-surgical recovery phase and the commencement of dialysis. An in-depth examination of the patient’s physiological metrics at numerous intervals was conducted to gain insights into disease evolution and the effectiveness of therapeutic interventions. Notably, the occurrence and intensity of hypotension and anemia among dialysis recipients were closely monitored, with a comprehensive evaluation of these conditions undertaken.

### 2.3 Literature review data sources and retrieval strategies

A review of relevant literature was conducted to enhance comprehension and examine the matters of hypotension, anemia, and postoperative tumor recurrence in hemodialysis patients after bilateral nephrectomy. This review extended to publications available in the MEDLINE, Embase, and Cochrane databases up to September 2023, utilizing “Carcinoma, Renal Cell,” “Nephrectomy,” and “Renal Dialysis” as principal search terms. The literature review aimed to supplement the empirical data with a broad scholarly context, thereby enriching the analysis with a comprehensive perspective on the studied phenomena.

### 2.4 Literature screening and quality assessment

Studies were incorporated based on specific inclusion criteria: (1) the study population comprised individuals diagnosed with renal carcinoma, (2) the intervention involved bilateral nephrectomy as the treatment modality, and (3) the primary outcome assessed was the incidence of adverse events (e.g., hypotension, anemia, or tumor recurrence) during postoperative hemodialysis. Exclusions applied to cellular and animal research, reviews, and meta-analyses. The literature screening process was facilitated using EndNote X9 software (Clarivate Analytics, USA), where duplicates were removed through both automated and manual efforts. Title, abstract, and full-text screenings were independently conducted by two researchers, adhering to pre-established eligibility criteria [28], with any conflicts resolved through consultation with a third researcher [29]. Preference was given to studies with larger participant samples in cases of population overlap. Consent forms were duly obtained from all participants. Additionally, the methodological quality of selected studies was independently evaluated by two researchers using Review Manager v5.4, with any discrepancies settled through third-party consultation [30].

### 2.5 Data extraction and analysis

Data from qualifying studies were independently extracted by two authors for subsequent analysis. Information gleaned included study design, geographic location of the study, year of publication, and participant demographics such as participant count, age distribution, gender ratio, and incidences of low blood pressure, anemia, and tumor recurrence. In instances of multiple reports, the most current or comprehensive data were prioritized. Corresponding authors were contacted for additional insights where necessary. An analysis exploring the association between hypotension, anemia, the use of ESAs, and renal cancer recurrence was conducted utilizing the MrBase platform (http://app.mrbase.org/).

### 2.6 Statistical analysis

Statistical analyses were conducted employing IBM SPSS software (USA). Descriptive statistics were applied to all continuous variables, with mean values and standard deviations calculated from a minimum of three independent experiments. The Student’s *t*-test was utilized to compare two datasets in case study analyses, while one-way analysis of variance was employed to compare across multiple groups in the review of literature-derived data. Nonparametric tests were applied in scenarios not meeting normal distribution or variance homogeneity prerequisites. Qualitative data, such as clinical symptoms and medical record signs, were analyzed using the chi-square test. All analytical tests were executed with a bidirectional approach to uphold analytical precision and reliability. Statistical significance was ascertained with a *p*-value threshold set at <0.05.


**Ethical approval:** This study rigorously followed international and domestic ethical guidelines and regulations to fully protect patients’ rights and welfare during the entire research process. The report does not include identifiable patient information, such as their name, address, or contact details. Patients and their families were provided with a comprehensive explanation of the study’s purpose and potential impacts, allowing them sufficient time to decide on their participation. The family members were informed of their right to withdraw their consent without incurring any negative consequences.

## 3 Results

### 3.1 Clinical patient medical records and analysis of this institute

A 57-year-old male patient was admitted to the hospital in 2009 due to bilateral lower back pain persisting for over 3 months. The patient experienced bilateral lower back pain 3 months ago without an obvious cause. The pain manifested paroxysmally and was accompanied by irritative symptoms related to the urinary tract but without hematuria. Physical examination revealed mild tenderness in both renal regions, with no evident positive signs identified elsewhere. All necessary examinations were conducted, and the diagnosis of “left renal clear cell carcinoma” was determined. A radical nephrectomy was then performed to treat the left renal cancer. The patient developed hematuria in June 2020, which led to the diagnosis of renal cancer in the right kidney. Subsequently, the patient underwent a radical nephrectomy for the right renal cancer, followed by post-operative hemodialysis treatment three times a week. The patient had a 10-year history of hypertension, with a recorded blood pressure of 180/100 mmHg. Oral medication was administered to lower blood pressure, resulting in a current reading of approximately 130/80 mmHg. After surgery, the patient recorded a pre-dialysis blood pressure of 150/90 mmHg, and no antihypertensive medications were given.

Six months after undergoing bilateral nephrectomy, a patient began experiencing frequent episodes of hypotension during the dialysis process, primarily occurring approximately 1 h after the initiation of the session. The patient’s blood pressure reached its lowest reading at 50/30 mmHg and was accompanied by symptoms of dizziness, chest tightness, and fatigue. The patient was prescribed oral Mido Jun (Midorijun) and Shengmai oral solution to increase sodium intake. Symptomatic treatments entailed high-sodium dialysis or adopting an adjustable sodium mode, low-temperature dialysis, blood volume regulation, and increasing dry weight. Nonetheless, the patient exhibited an unsatisfactory response to the treatment. During dialysis, the patient received dopamine to elevate blood pressure and hypertonic saline to sustain blood pressure. The patient experienced persistent low blood pressure along with severe anemia. Before the bilateral nephrectomy, the patient’s hemoglobin level was 123 g/L, gradually decreasing to 110 g/L after dialysis. A weekly dosage of 10,000 units of ESA (EPO) was administered, and before the completion of each dialysis session, 5 mL of sucrose iron was infused. The patient’s hemoglobin decreased and reached 61 g/L in November 2021. Subsequently, the patient received an oral dose of rosuvastatin 120 mg thrice weekly, gradually increasing their hemoglobin levels until they returned to normal. During the examination in March 2022, multiple masses were detected in the patient’s liver and lungs. By then, the blood pressure and hemoglobin levels had already returned to near-normal. The patient succumbed to cachexia caused by liver cancer and passed away in June 2022.

### 3.2 Case presentation

Despite losing the kidneys’ ability to detoxify and regulate electrolyte and acid–base balance in dialysis patients, the kidneys remain crucial in maintaining blood pressure. The kidneys primarily regulate blood pressure stability through the renin–angiotensin–aldosterone system (RAAS). Renin is predominantly secreted by juxtaglomerular cells in the renal glomerulus. Nonetheless, research has demonstrated that renin can also be synthesized in diverse tissues and organs, including the adrenal glands, heart, brain, pancreas, and adipose tissue, constituting the tissue renin–angiotensin system (RAS) [31,32].

The blood pressure conditions of four maintenance hemodialysis patients who had undergone bilateral nephrectomy for 2–8 years were observed at China–Japan Friendship Hospital. Two cases demonstrated a rapid onset of hypotension following resection, with a sustained hypotensive state lasting 2–6 years. The patient’s RAS blood test revealed a renin activity level of 0.08 ± 0.03 ng/mL, significantly below the normal range of 0.93–6.56 ng/mL. The levels of angiotensin II and aldosterone in the blood fall within the normal range, with measurements of 71.37 ± 8.28 pg/mL and 0.17 ± 0.02 ng/mL, respectively. One possible reason is that the kidneys, as the primary organ responsible for producing renin, substantially reduce renin levels following their removal.

In contrast, the RAS system in other organisms regulates angiotensin II and aldosterone levels by gradually secreting renin, thereby maintaining a state of vascular tension. During dialysis, dehydration causes the removal of components of the RAAS system, leading to delayed production of renin by the body and subsequent hypotension. After approximately 2 years, the RAS compensates by increasing its secretion, resulting in the recurrence of hypertension in the patient. Furthermore, renin alone does not account for the production of AngII. When there is an early deficiency of renin, the RAS levels decrease, leading to a reduction in blood pressure. Nevertheless, the generation of AngII, independent of renin, gradually rises, which enables the levels of AngII and aldosterone in the bloodstream to normalize and, eventually, restore blood pressure, potentially even increasing it.

### 3.3 Older patients who have undergone bilateral nephrectomy may carry a greater risk of cancer recurrence

**Figure 1:**
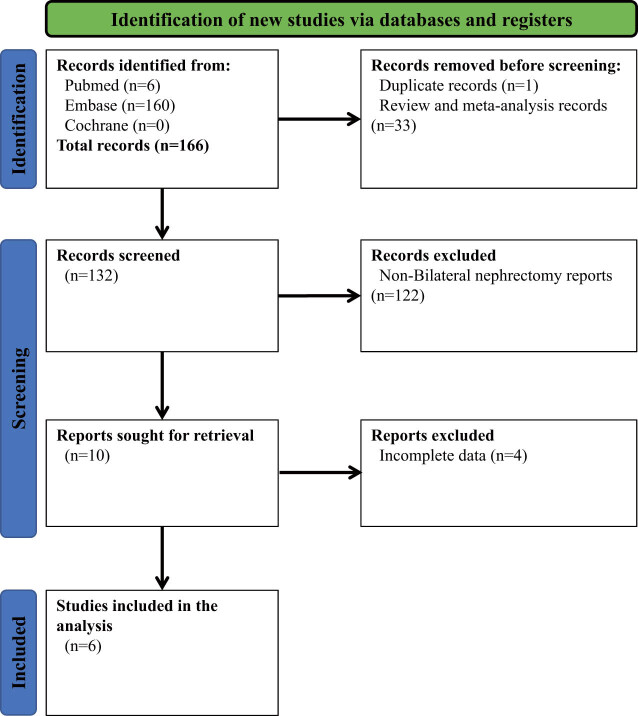
PRISMA 2020 flowchart.

**Figure A1:**
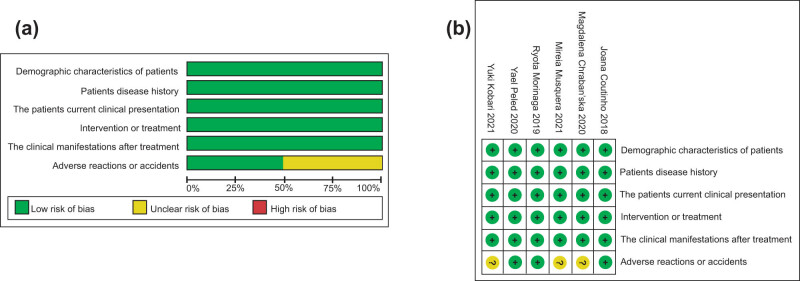
Quality assessment of included studies. Note: (a) Risk of bias graph, summarizing the judgment for each bias risk item across all included studies as a percentage. (b) Risk of bias summary, providing an overview of the judgment for each bias risk item in each included study, with red indicating high risk, green indicating low risk, and yellow indicating unknown risk.

To further investigate the risk factors associated with tumor recurrence in patients undergoing bilateral nephrectomy, we conducted a search and compilation of relevant literature. Figure 1 illustrates the summary of the process of literature retrieval and selection. One hundred sixty-six articles were retrieved by referencing the MEDLINE, Embase, and Cochrane databases. Subsequently, a step-by-step screening process excluded the literature unrelated to bilateral nephrectomy and had incomplete data. Finally, a total of six case reports were obtained for analysis. No additional articles that met the criteria were identified during the thorough examination of the reference list of the included studies. All the articles included in this study have complete texts and have been published in peer-reviewed journals. Please refer to Figure A1 for the assessment of the quality of the literature. After conducting a quality assessment, it was determined that all studies included in the field of indicator selection had a low risk of bias.

Table 1 presents a summary of the baseline characteristics that were included in the study. The six studies included seven kidney cancer patients who underwent bilateral nephrectomy as a treatment. The research comprised three studies, representing 50% of the total, conducted in Europe (Poland, Portugal, and Spain). Additionally, three studies representing 50% of the total were conducted in Asia (Japan and Israel). The study included predominantly middle-aged and elderly men aged 35 and above. Three of the six studies included showed evidence of tumor recurrence in patients following surgery. Among these cases, two patients had papillary renal cell carcinoma and clear cell carcinoma, while one patient had chromophobe cell carcinoma. Additionally, only one study documented the occurrence of hypotension in patients post-surgery.

**Table 1:** Baseline characteristics of patients included in the study

Study	Country	Sample size (% female)	Age (years)	Hypotension	Anemia	Tumor recurrence (%)	Pathology
Magdalena Chrabańska et al. (2020)	Poland	1 (0)	45	—	—	0	ACD-RCC
Joana Coutinho et al. (2018)	Portugal	1 (100)	29	—	—	0	Chromophobe renal carcinoma
Yuki Kobari et al. (2021)	Japan	1 (0)	63	—	—	100	L: Papillary
Ryota Morinaga et al. (2019)	Japan	1 (0)	68	—	—	100	R: Clear cell
Mireia Musquera et al. (2021)	Spain	1 (0)	53	—	—	100	Clear cell + papillary
Yael Peled et al. (2020)	Israel	2 (0)	36 (35–37)	0	—	0	Chromophobe renal carcinoma

Analysis of six publications [33–38] revealed a 100% probability of tumor recurrence after bilateral nephrectomy in elderly patients (age >50 years) with renal cancer, whereas no tumor recurrence was observed in middle-aged patients. It suggests a significantly higher risk of tumor recurrence in elderly patients following surgery (*P* = 0.0057). Considering the evident occurrence of hypotension and anemia in our post-surgical patients, we have also gathered postoperative performance data from patients mentioned in the literature. However, current research reports lack relevant records regarding hypotension and anemia during hemodialysis in patients who have undergone bilateral nephrectomy for renal cancer. To investigate the potential association between hypotension, anemia, and tumor recurrence in patients undergoing bilateral nephrectomy, we analyzed the relationship between these factors and the occurrence of renal cancer using the MrBase website. Nevertheless, our findings demonstrate no direct causal link between either hypotension or anemia and the development of kidney cancer or tumor formation.

### 3.4 Correlation between the increased tumor recurrence rate in patients after bilateral nephrectomy and drug treatment

In our research, we observed a worrying phenomenon of tumor recurrence among patients in our institution following the administration of ESAs and rosuvastatin. This finding motivated us to conduct additional research on the possible association between these two medications and the recurrence of tumors. To better understand this relationship, we consulted previous literature and discovered several studies supporting prolactin’s potential role in promoting tumor recurrence [39,40].

To investigate this potential relationship further, we employed the resources of the MrBase website to seek additional evidence regarding the link between ESAs and tumor recurrence. However, insufficient data is available on the website to establish a definitive association between ESAs and kidney cancer or the recurrence of other cancers. Moreover, the limited number of reported cases restricted our ability to establish a correlation between post-bilateral nephrectomy treatment and tumor recurrence from the existing literature. The absence of this particular data may indicate the challenges associated with conducting research in this field and our limited understanding of the potential relationships at this stage.

## 4 Discussion

Hypotension represents a prevalent complication among dialysis patients, manifesting through symptoms like chest tightness, dizziness, and nausea, and in severe instances, can lead to consciousness alterations and insufficient dialysis, thus significantly elevating mortality risk [41]. Despite its frequency, there lacks a universally accepted definition for hypotension in the context of dialysis, with criteria varying from minimum systolic blood pressure thresholds to the necessity for clinical interventions [42–44]. Clinically, hypotension during dialysis is often characterized by a reduction in mean arterial pressure exceeding 30 mmHg or a drop in systolic blood pressure below 90 mmHg, irrespective of symptomatic presentation [45,46]. This study elucidates the case of a hypertensive patient experiencing a progressive decline in blood pressure following bilateral nephrectomy, underscoring the critical role of the kidneys in blood pressure regulation even in patients undergoing dialysis.

The analysis of six studies reveals that elderly patients (age >50) had a 100% probability of tumor recurrence after bilateral nephrectomy for renal cancer. In contrast, no tumor recurrence had been observed in middle-aged patients, suggesting that elderly patients face a higher risk of tumor recurrence post-surgery. This high incidence indicates an elevated level of physical stress experienced by patients during dialysis and potentially suggests the occurrence of other complications. The higher recurrence rate in elderly patients may be attributed to factors such as their overall health condition and the complexity of the dialysis process. It is also important to consider the patient’s age and the time elapsed since the double kidney removal, as these factors may influence the outcome. Further research is needed to conduct a more detailed data analysis to understand this phenomenon and its relationship with other variables fully.

In the case reported in this study, the patient underwent bilateral nephrectomy and received treatment with 10,000 units of ESA (EPO) per week for an extended period. Given the anemia-related parameters indicative of iron deficiency, intravenous sucrose iron at a dosage of 5 mL was administered prior to the conclusion of dialysis treatments. Despite these interventions, the patient’s anemia continued to deteriorate, with hemoglobin levels reaching a nadir of 61 g/L. Consequently, oral rosuvastatin at a dose of 120 mg, administered thrice weekly, was introduced. This treatment regimen led to a gradual increase in hemoglobin levels, ultimately returning to within the normal range. The therapeutic use of rosuvastatin and ESAs, which promote red blood cell proliferation and bone marrow hematopoiesis, represents a primary approach in managing renal anemia. However, our literature review has identified a potential association between the use of ESAs and an elevated risk of tumor recurrence, aligning with findings from previous studies such as the ENHANCE trial and the BEST study. These studies suggest that while high doses of ESAs can elevate hemoglobin levels, they may also expedite tumor progression [47]. The clinical practice incorporates three generations of ESAs: the first generation, recombinant human erythropoietin, is a sialoglycoprotein hormone with immunological and biological characteristics highly similar to endogenous human erythropoietin, yet with a short half-life requiring administration one to three times weekly. The second-generation ESA, darbepoetin alfa, features two glycosyl chains connected to its N-terminus, offering increased metabolic stability *in vivo* due to its highly glycosylated structure. The third-generation ESA, continuous erythropoietin receptor activator (CERA), a chemically synthesized CERA, presents a unique mechanism of action. Compared to currently available recombinant human erythropoietin, it offers advantages such as a longer half-life (approximately 130 h via intravenous or subcutaneous injection) and less frequent dosing requirements [48]. While ESAs demonstrate efficacy in treating anemia, their potential risk in increasing tumor recurrence cannot be overlooked.

Rosuvastatin is known to stimulate erythropoietin production, yet the effectiveness of this mechanism is questioned following the removal of the kidneys, which are primary erythropoietin producers [49–51]. While the liver predominantly produces erythropoietin during fetal development, postnatal erythropoietin synthesis shifts to the kidneys. Nevertheless, alternative sites such as the liver, brain, and bone marrow can produce erythropoietin, regulated by hypoxia-inducible factors [52,53]. In the context of bilateral nephrectomy, rosuvastatin acts on these hypoxia-inducible factors across various organs and tissues, offering a strategic approach to anemia management by promoting erythropoietin synthesis outside the kidneys [50,51,54]. Moreover, rosuvastatin has been found to surpass erythropoietin in reducing micro-inflammation, combating erythropoietin resistance, and improving iron utilization [55,56]. Although current animal studies indicate that roxadustat does not promote the occurrence, progression, or metastasis of VEGF-sensitive NeuYD mouse tumors [57,58], there is limited data on its effect on human tumor incidence. In our reported clinical case, we observed tumor recurrence in a patient who underwent bilateral nephrectomy and received ESA treatment. Analysis of our reported and collected cases, although limited, suggests a weak statistical correlation between tumor recurrence and ESA treatment. However, given the significant impact of tumor recurrence on the prognosis of patients who have undergone bilateral nephrectomy, this clinical phenomenon warrants further attention and research. Therefore, it is essential to investigate whether the dosage of roxadustat and erythropoietin should be reduced once hemoglobin levels are normalized in patients with renal tumors undergoing bilateral nephrectomy.

Postoperative dialysis necessity is notably low following bilateral renal tumor resection, with previous research suggesting limited efficacy of hypotension treatments in this population. The primary goal of such treatments is to mitigate the discomfort caused by hypotension, with observation posited as a viable strategy in symptom-free cases. Typically, blood pressure normalization is achieved within 2–4 years post-treatment. Additionally, bilateral nephrectomy patients may develop anemia, for which Rosacetra has been identified as a potent stimulator of erythropoietin production by the liver and other organs, facilitating erythropoiesis [59,60]. In patients with tumors, the use of erythropoietin and other treatments for anemia should take into account the potential risks of tumor recurrence and accelerated progression. Physicians should communicate thoroughly with patients to avoid medical disputes. For patients undergoing bilateral nephrectomy for renal tumors, early kidney transplantation is recommended to prevent adverse effects such as hypotension.

In addition to the limited number of cases available for study, there are many confounding factors that need further exploration in the analysis of the association between ESAs and renal cancer recurrence. These factors include tumor pathology type, tumor staging, and the presence of lymph node metastasis [61]. The information provided by the MrBase website and the included case reports is not comprehensive enough to allow for detailed analysis of these potential confounders. Tumor recurrence is often the result of complex interactions regulated by a multifaceted network, involving multiple genes and factors, which adds to the complexity [62,63]. Therefore, analyzing the potential confounding factors in tumor recurrence may require more complete data collection and an increased number of cases. This would help in establishing a more robust database to address these confounding factors effectively.

Although patients face numerous challenges following bilateral nephrectomy, kidney transplantation emerges as a promising avenue for addressing complications associated with dialysis and enhancing quality of life. However, it is important to recognize that the 5-year survival rate for patients with advanced kidney cancer remains subdued, influenced by a myriad of factors, including a shortage of donors, the complexity of surgical procedures, the misdiagnosis of kidney cancer, and the late detection of tumors [64]. Additionally, the body of literature contributing to our understanding of these issues is relatively small, and the available references often lack detailed records on the treatment modalities, variations in patient populations, dosages of ESAs, or other relevant clinical variables. This significant gap underscores the need for comprehensive data to better guide clinical decisions and policy formulations. These challenges contribute to the relatively rare occurrence of kidney transplants in patients who have undergone bilateral renal tumor resection, underscoring a pressing need for further research focused on identifying and implementing strategies to improve the feasibility and efficacy of kidney transplantation for these patients. Enhancing the scope and depth of research in this area could potentially transform the treatment landscape for patients with renal cancer, offering them a better prognosis and quality of life post-surgery.

**Figure 2:**
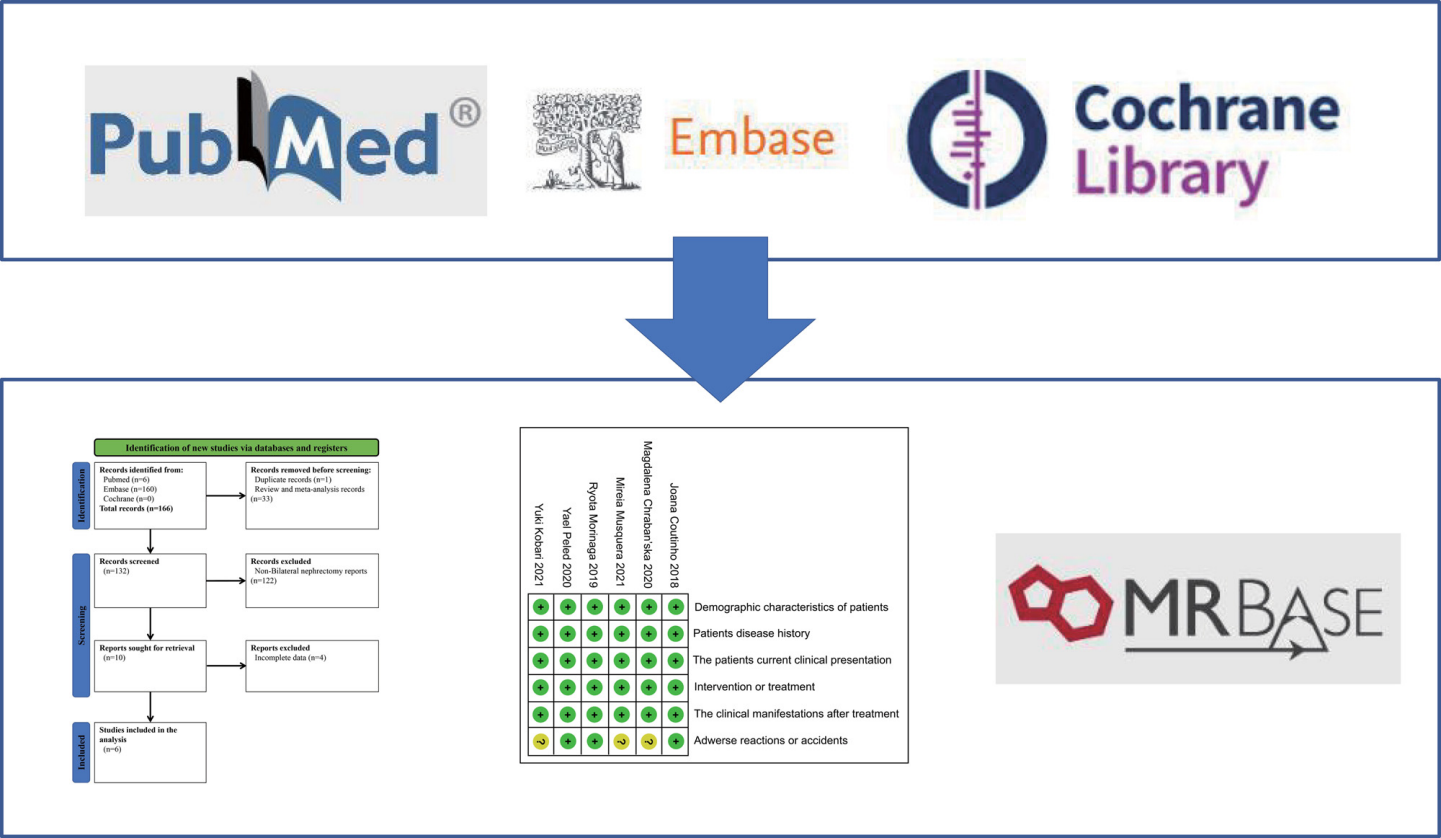
Diagram illustrating the relationship between risk factors such as hypotension and anemia during hemodialysis in patients after bilateral nephrectomy and tumor recurrence based on evidence-based medicine.

This study examines the issues the patient may experience while undergoing hemodialysis after bilateral nephrectomy and provides a review of existing literature to shed light on current knowledge and uncover gaps in our understanding (Figure 2). This study highlights the complexity of managing anemia with ESAs, cautioning against the potential for these drugs to induce or accelerate potential tumor recurrence and progression. The study suggests that early kidney transplantation might be the optimal strategy to circumvent adverse reactions such as hypotension that are associated with post-nephrectomy dialysis. However, the study’s insights are constrained by several limitations, notably the sparse and incomplete records of postoperative medication and the lack of utilization information for ESAs or statin drugs across the examined cases. Such limitations restrict a comprehensive examination of the relationship between these medications and tumor recurrence. The scarcity of case reports and the absence of detailed information on cancer subtypes or stages in the available literature limit the ability to perform a nuanced analysis of tumor recurrence across different renal cancer patients. Moreover, the statistical correlation between tumor recurrence rates, bilateral nephrectomy, and drug treatment is not strong due to the limited number of cases analyzed. The data from the MrBase website do not clearly support a link between erythropoietin and the recurrence of renal or other cancers. These limitations can be addressed in future studies with larger sample sizes and more comprehensive data collection. Addressing these gaps and prioritizing the conduct of additional clinical trials are essential steps forward. These efforts would improve our understanding and enhance the management of post-nephrectomy complications, paving the way for more effective and patient-centric care strategies in the aftermath of bilateral nephrectomy.
